# Heart rate variability, mood and performance: a pilot study on the interrelation of these variables in amateur road cyclists

**DOI:** 10.7717/peerj.13094

**Published:** 2022-03-30

**Authors:** Carla Alfonso, Lluis Capdevila

**Affiliations:** 1 Laboratory of Sport Psychology, Department of Basic Psychology, Universitat Autónoma de Barcelona, Barcelona, Spain; 2 Sport Research Institute, Universitat Autònoma de Barcelona, Barcelona, Spain

**Keywords:** Heart rate variability, Mood, Performance, Training load, Athletes, HRV

## Abstract

**Objective:**

The present study seeks to explore the relationship between measures of cycling training on a given day and the heart rate variability (HRV) and mood states obtained the following morning. The association between HRV and mood state is also studied, as is the relationship between internal and external measures of training.

**Methods:**

During a 6-week period, five recreational road cyclists collected 123 recordings of morning HRV and morning mood, and 66 recordings of training power and rate of perceived exertion (RPE). Training power was used as an external measure of performance and RPE as an internal measure of performance. The HRV parameters used in the study were the mean of RR intervals (mean RR) and the standard deviation of all RR intervals (SDNN) as time domain analysis, and the normalized high frequency band (HFnu), normalized low frequency band (LFnu) and the ratio between low and high frequency bands, as frequency domain analysis. Mood was measured using a 10-point cognitive scale.

**Results:**

It was found that the higher the training power on a given day, the lower the HFnu and the higher LF/HF were on the following morning. At the same time, results showed an inverse relationship between training and mood, so the tougher a training session, the lower the mood the following day. A relationship between morning HRV and mood was also found, so that the higher mean RR and HFnu, the more positive the mood (r = 0.497 and r = 0.420 respectively; *p* < 0.001). Finally, RPE correlated positively with external power load variables (IF: r = 0.545; *p* < 0.001).

**Conclusion:**

Altogether, the results indicate a relationship between training of cyclists on a given day and their morning HRV and mood state on the following day. Mood and HRV also seem positively related. It is argued that developing a monitoring system that considers external and internal training loads, together with morning mood, could help understand the state of the individual, enabling feedback to athletes to facilitate the adaptation to training and to prevent problems associated with overtraining. However, more research is needed to further understand the association between the different variables considered.

## Introduction

To build fitness, an individual needs to apply a stress to the body, and then through recovery, the body adapts and is able to accommodate greater stress in the next round of training. Strategies to control the adequate amount of stress and recovery are essential to facilitate the adaptation of athletes to training and to ameliorate performance (Morales et al., 2014; Wallace, Slattery & Coutts, 2014), as well as to prevent problems associated with overtraining, including injuries (Owen et al., 2015; Rodas et al., 2008).

Physical stress, or training load, is the dose of training completed by an athlete during an exercise bout and is in part responsible for fitness gains. Too much of it can result in overtraining and loss of fitness, whereas too little can result in no improvement. Training load can be quantified by a variety of methods, divided into external or internal. External methods include measures about the training itself, such as distance, speed or power and their recording is facilitated by the emergency of gadgets such as GPS devices (Buchheit et al., 2010; Wallace, Slattery & Coutts, 2014). In cycling, Normalized Power (NP) is the most used reading. NP is measured in watts and it is the estimate of the force that the individual can maintain for a given physiological cost if this force was constant during a given period of time (Wallace, Slattery & Coutts, 2014). Other external metrics are the intensity factor (IF) and training stress score (TSS), which estimate the overall physiological stress created by a training session, and which have been reported appropriate for monitoring and quantifying training load in cycling (Wallace, Slattery & Coutts, 2014).

Internal methods record the athlete’s response to training and include biomarkers such as heart rate (HR), as well as subjective questionnaires including the rating-of-perceived-exertion (RPE) (Lambert & Borresen, 2006). RPE is a well-known and accepted scale for monitoring training, as it incorporates the relative psychophysiological stress imposed on the athlete (Foster et al., 2001; Viru & Viru, 2000). Apart from HR, another internal biomarker which has been gaining attention in the last decade is heart rate variability (HRV) (Plews et al., 2013). HRV is the variation in heartbeats (RR interval) within a specific timeframe and within the analysis of consecutive circadian periods, and can be measured by time or frequency domain methods. The statistical analyses of the length dispersion of RR intervals around a mean is the basis of time-domain indices. Differently, the spectral analysis of different components of the RR curve is the basis of frequency-domain parameters (Xhyheri et al., 2012). In medical research, HRV is being used as an objective and non-invasive tool to assess cardiac autonomic activity (TFESC, 1996) and in the athletic context, HRV is thought to serve as a marker for the adaptation of athletes to training (Kiviniemi et al., 2007; Rodas et al., 2008). HRV monitors the autonomic control on the cardiac system, describing the capacity of the organism, and specifically of its cardiovascular system, to alter the HR beat to beat to adapt to external and internal demands such as those imposed by training (Morales et al., 2014; Ortega & Wang, 2018). Previous studies show that when training load increases, HRV, measured in the morning, once a day, decreases. A reduction in resting HRV, has been related to fatigue, overtraining and the inability of the cardiovascular system to adjust to increasing levels of training, resulting in reduced performance (Dishman et al., 2000; Garet et al., 2004). Conversely, increase in performance, for example during competition, corresponds to positive changes in sympathetic and vagal activity (Buchheit et al., 2010; Cervantes, Rodas & Capdevila, 2009; Ortigosa-Márquez et al., 2017).

At the same time HRV has also been related to emotional responding. Changes in the Autonomic Nervous System (ANS) are reflected in HRV and are thought to indicate the ANS’s ability to adjust arousal of an individual to the demands of the environment. Wheat & Larkin (2010) showed that high HRV responds to activation of the ANS to suit the demands of the stressful situation, whereas low HRV shows poor responsiveness of the ANS. In the general population, high HRV is related to more context-appropriate motivational responses, better executive attention and better working memory performance (Gillie, Vasey & Thayer, 2014) than low HRV, and it has also been related to reported feelings of relaxation amongst healthy participants (Lin, Tai & Fan, 2014). In the area of sports, low levels of HRV are associated with psychoemotional states including anxiety and a difficulty to face competition (Kindermann, 1986; Kleiger, Stein & Bigger, 2005; Lee, Wood & Welsch, 2003). When using the profile of mood states (POMS) questionnaire, Vigor and Fatigue subscales strongly correlated to changes in HRV, in particular to changes in the LF/HF balance (Moreno, Parrado & Capdevila, 2013). Moreover, altered Fatigue scores on the POMS also correlated to symptoms of overtraining or staleness, suggesting that emotional state could in turn be an indicative of athletic performance (Moreno, Parrado & Capdevila, 2013).

In summary, there are two main approaches to assess the amount of stress and recovery that athletes undergo: external methods give an insight into the characteristics of the training sessions, whereas internal methods inform about the physical and psychological responses of the individual to that session. Multiple studies suggest that measures of both internal and external methods relate to one another, as well as with emotional states. Understanding these associations is relevant to monitor training and to determine the balance of stress-recovery that each athlete needs to improve performance and prevent injuries. Following this line of research, the present study seeks to investigate the relationship between the psychophysiological state of cyclists in the morning and their training from the previous day. It also investigates the relationship between HRV and mood from the same day, and between internal and external training data. In particular, the study will look into the relationship between morning HRV, as indicator of the physiological condition of the athletes, morning mood, as indicators of their psychological state, and the previous day’s training power output and RPE, as external and internal indicators of performance, respectively.

## Materials and Methods

### Sample

Five recreational road cyclists volunteered to participate in this study. All were males with an average of 31.6 ± 3.4 years, had at least 6 years of experience riding and completed at least two training sessions per week for the duration of the study. Each cyclist was fully informed of potential risks and benefits associated with participation. Written informed consent was obtained from each participant. The study was conducted according to the Local Ethics Commission for Human Experimentation of the Autonomous University of Barcelona (protocol code CEEAH-5745).

### Instruments

#### Anthropometric data and training indices

Age, height, weight and body mass index (BMI) were collected as anthropometric data. Functional threshold power (FTP) and watts/kg were used as fitness indices. This data was provided by the cyclists at the beginning of the study.

#### Mood state

Mood was measured upon waking up using a 10-point cognitive scale, as has been used in other recent studies (Perez-Gaido et al., 2021). Cyclists responded to the question “How do you feel right now” (1–sad to 10–happy).

#### HRV recordings

Upon waking up, and prior to any movement or ingest, participants were asked to remain in supine position in bed and record HRV data continuously for 3 min of natural breathing. Participant’s beat-to-beat cardiac intervals (RR) were recorded using a Polar H7 chest band (Polar Oy, Polar Electro, Kempele, Finland). The accuracy and reliability of the Polar Band H7 was previously tested with the gold standard based on the ECG (Parrado et al., 2010). RR intervals with a resolution of 1 ms were sent by Bluetooth (BTv4) to the FitLab App (HealthSportLab.com, Barcelona, Spain), which was downloaded into a mobile device (iOS, Apple) in order to record the RR series. This application was connected *via* wireless to a remote server for analysing HRV parameters. The system allows performance of individual HRV recordings in each session and checks data quality in real time.

#### External training load

External training load was studied using normalized power (NP), intensity factor (IF) and training stress score (TSS). NP is an estimate of the power that the athlete can maintain for a physiological cost if power is constant during a given period of time. IF is the ratio of the NP to the rider’s functional threshold power. It gives a relative intensity in relation to threshold power, making IF a convenient way of comparing the relative intensity of training within or between riders. TSS is calculated based on IF and takes into account the intensity, duration, and frequency of a workout to estimate the overall stress created by that training session, as defined by TrainingPeaks (TrainingPeaks, Louisville, KY, USA). The single value of IF or TSS can represent how hard an athlete worked out, with the higher the numbers, the harder the training session. TSS and IF allow to quantify and compare workout between athlete because these values are relative to each individual’s threshold, so that a 100 TSS points earned by a pro is relatively the same as 100 TSS points earned for a beginner (TrainingPeaks, Louisville, KY, USA).

All data was recorded during training sessions and obtained through each cyclists’ TrainingPeak’s profile. Power data was obtained using a power meter Assioma Duo (Favero Electronics SRL, Italy).

#### Internal training load

Internal training load was studied using the Rate of Perceived Exertion (RPE) scale to measure perceived effort. The athletes had to rate the intensity of the entire training session using a category ratio scale CR10 of Borg (1985), from 1 to 10, where “1” corresponds to no exertion, and “10” to maximal exertion. RPE data was taken 30 min following the completion of the cycling session to ensure the cyclists reported perception for the entire training session.

### Procedure

A longitudinal case study design was used to compare HRV and performance responses of the volunteers during a 6-week period. All eligible participants attended a first face-to-face interview individually in which demographic and fitness indices were collected. The protocol of the study was explained. After the first interview, two sets of data were recorded, according to the individual training planning of each cyclist:

a) Training data: during the 6-week period, each participant trained individually, following their own training plan, which consisted of riding road bikes at least twice a week. The study didn’t interfere with their training routine. For each training session, power data was recorded and was used to calculate TSS and IF using a power meter. RPE was also recorded. Data was saved and processed using the app TrainingPeaks.
b) Morning data: After days of training, at home on their own, upon waking on the morning, in a supine position in bed, and prior to any movement or ingest, each participant answered a 10-point mood scale. Immediately afterwards, maintaining the supine position in bed, each participant was required to complete a 3 min HRV test. Data was collected and analysed with the FitLab® system (HealthSportLab.com, Barcelona, Spain).

Participants were asked to complete the maximum number of training sessions per week possible, followed by recording morning data the day after. A total of 123 morning registers (mood and 3 min HRV test) and a total of 66 training registers were recorded. A total of 57 HRV morning recordings were not preceded by a road training the day before. During the 6-week study period, each participant completed an average of 25 morning registers (4 per week) and 12 training registers (2 per week).

### HRV data analysis

HRV analysis was performed for RR intervals in 3 min periods, as carried out in other field studies where it was required to interfere as little as possible in the training routines of athletes (Martín-Guillaumes et al., 2018). Ultra-short-term HRV analysis (30 s or 60 s) was recommended at rest condition (Wu et al., 2020), with a minimum of 120 s needed to record low frequency components (Munoz et al., 2015).

HRV data was obtained from the FitLab system. All RR intervals obtained were filtered and records exceeding an error rate of 11% were not considered for analysis. The mean of signal error for all recorded RR was 0.91%. Matlab’s scripts (MathWork, Portola Valley, CA, USA) were used for error correction, as developed and validated in previous publications (García-González et al., 2015; Parrado et al., 2010). The error correction of RR series was applied as defined by García-González et al. (2015). HRV analyses were performed following the recommendations of the Task Force of the European Society of Cardiology and the North American Society of Pacing and Electrophysiology (TFESC, 1996). HRV parameters were calculated as defined by Escorihuela et al. (2020): for time domain analyses, mean of RR intervals (mean RR) and the standard deviation of all RR intervals (SDNN) were calculated, and for frequency domain analysis the power in the low frequency (LF) band (0.04–0.15 Hz) and the high frequency (HF) band (0.15–0.40 Hz) were used. Additional calculations included the normalized LF and HF values (LFnu and HFnu, respectively), and LF/HF parameters.

### Statistical analysis

Analyses were carried out with all recordings for the five cyclists. Descriptive data are presented as mean ± SD. Pearson correlation analyses were performed in order to show bivariate relationships between HRV, mood, RPE, and external training load parameters. Multiple regression analysis was applied for explaining mood and HRV as a function of training parameters from the previous day, as well as mood as a function of resting HRV parameters in the same morning session. The regression method used was stepwise. G*Power (v3.1; Heinrich-Heine-Universität Düsseldorf, Düsseldorf, Germany) was used to analyse statistical power and effect size for regression analysis (Faul et al., 2009). Other statistical analyses were performed using IBM SPSS Statistics Package for Mac OS (version 28.0; SPSS Inc., Chicago, IL, USA). Statistical significance was set at *p* < 0.05. All data are presented as mean ± SD unless otherwise stated.

## Results

### Description of anthropometric data and training indices

The cycling group consisted of five male recreational road cyclists. Anthropometric characteristics and training indices are shown in Table 1.

**Table 1:** Anthropometric characteristics and training indices.

	P1	P2	P3	P5	Mean	SD
Age	35	34	29	27	31.25	3.3
Height	180	172.5	179	186	179.38	4.8
Weight	74	62	64	68	67	4.6
BMI	22.8	21	20	19	20.7	1.4
FTP	330	242	340	325	309.25	39.2
Watts/kg	4.46	3.90	5.31	4.78	4.61	0.5

**Note:**

Summary of participant’s anthropometric data and training indices. P1, P2, P3, P4, P5 stands for participants 1, 2, 3, 4 and 5 respectively. Height is expressed in centimetres; weight is expressed in kilograms; Body Mass Index (BMI) is expressed in kilogram by heigh in meters squared (kg/m^2^); Functional Threshold Power (FTP) is expressed in watts; resting HR is expressed in beats per minute. Average data is expressed as mean (SD).

### Description of the main parameters analysed in the study

Table 2 shows means and standard deviations for the parameters analysed in the two situations under study, Training and Morning.

**Table 2:** Means and standard deviations for all training and morning parameters.

Morning data (*n* = 123)	RRmean	SDRR	LF/HF	LFnu	HFnu	Mood
Mean (SD)	1,262.46 (179.3)	118.16 (36.3)	1.40 (1.3)	51.61 (16.6)	48.39 (16.6)	5.80 (2.5)
**Training data (*n* = 66)**	**NP**	**TSS**	**IF**	**RPE**		
Mean (SD)	189.35 (40.1)	120.48 (83.86)	0.65 (0.2)	5.50 (2.2)		

### Correlation coefficients and regression analyses

The following tables show the correlation between variables registered in the morning and those during training completed on the previous day.

## Mood and HRV as a function of yesterday’s training

Table 3 illustrates the correlation between external training load and the following morning’s general emotional state and HRV variables. The training variable IF negatively correlated with Mood (r = −0.416; *p* < 0.01) and HFnu (r = −0.421; *p* < 0.01), and positively with LH/HF (r = 0.256; *p* = 0.034) and Lfnu (r = 0.421; *p* < 0.01). A multiple regression analysis was performed, but there was no significant equation that explained resting HRV or mood from a lineal combination of training parameters from the previous day. All significant relationships between variables can be explained by simple correlations illustrated in Table 3.

**Table 3:** Pearson correlation coefficients between morning parameters (mood and HRV), and training variables from the day before (*n* = 66 recordings).

	NP	TSS	IF	RPE
Mood	−0.259	−0.091	−0.416**	−0.062
RRmean	−0.171	−0.201	0.041	0.276*
SDRR	0.153	0.086	0.270*	0.357**
LF/HF	0.264*	0.302*	0.256*	0.156
LFnu	0.385**	0.299*	0.421**	0.212
HFnu	−0.385**	−0.299*	−0.421**	−0.212

**Notes:**

* Significant correlation at 0.05 (bilateral).

** Significant correlation at 0.01 (bilateral).

**Figure 1:**
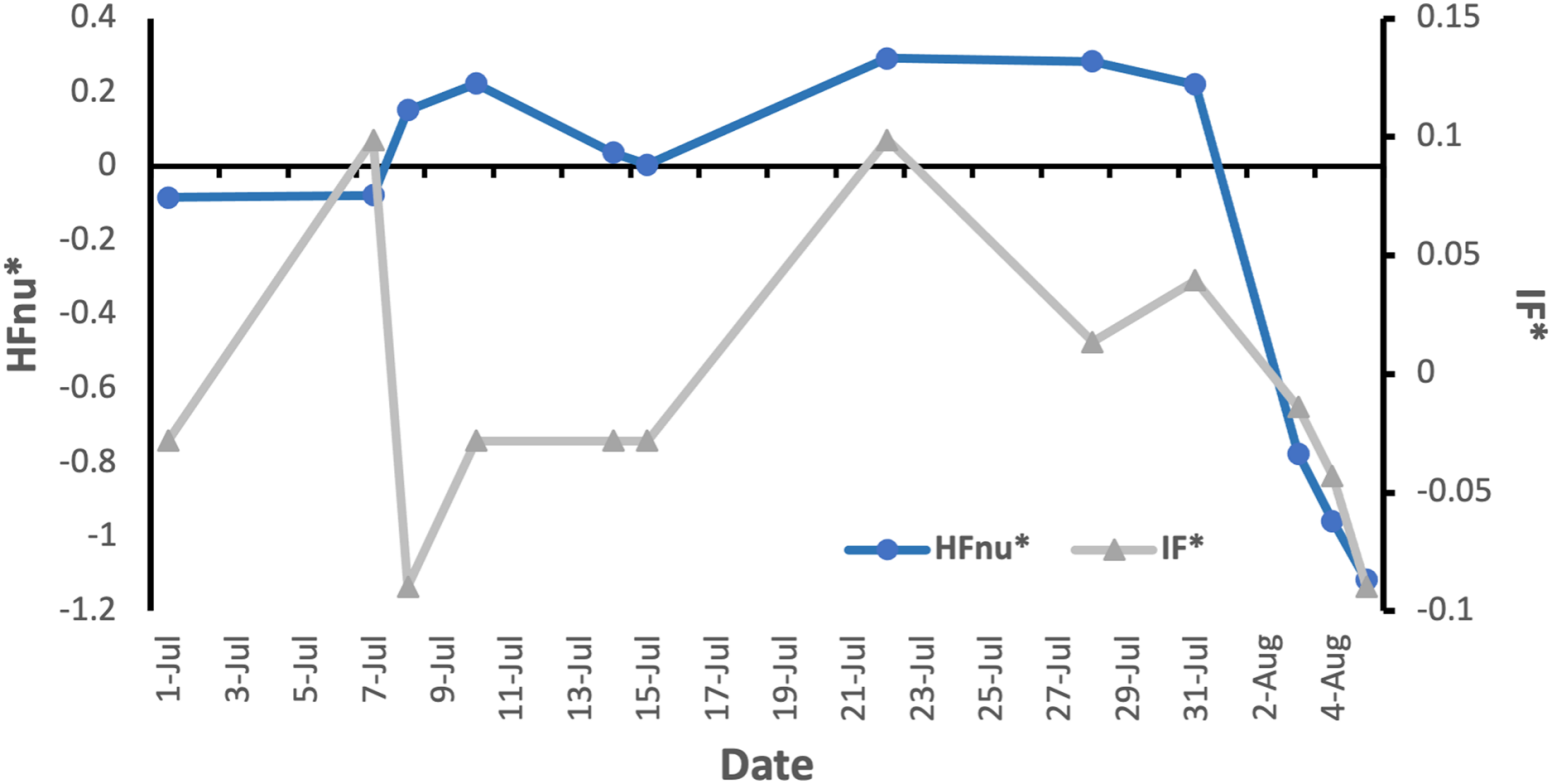
Representation of data points obtained from Participant 1. The graph illustrates the relationship between external training load (IF) and cardiac variability the next morning (HFnu), for 12 training recordings obtained from this participant. HFnu* = (HFnu − 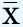
HFnu)/HFnu; IF* = (IF − 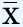
IF)/IF.

Figure 1 illustrates a case example of the relationship between external training load (IF) and cardiac variability the next morning (HFnu). IF and HFnu are chosen as the external load and HRV parameters, respectively, because they showed the highest correlation between sets of variables (Table 3). In Fig. 1, data points are represented in relation to the overall average values obtained from the participant in question.

## Morning mood and HRV

Table 4 shows that general emotional state, as recorded by Mood, significantly correlates with all HRV parameters registered in the same morning session (*p* < 0.001, in all cases). Moreover, Mood was studied as a function of HRV indices, and the resulting model shows that morning mood can significantly be explained by a lineal combination of RRmean, SDNN and Hfnu (Table 5). This combination of HRV parameters explains 49.2% of the mood variability. The effect size (f^2^ = 0.968) is large for a R^2^ = 0.492, *n* = 123, and three predictors (f^2^ > 0.35). In the same way, the statistical power (*π* = 1.00) is also large (*π* > 0.80; Faul et al., 2009).

**Table 4:** Pearson correlation coefficients between morning mood and HRV parameters from the same session (*n* = 123 recordings).

	RRmean	SDRR	LF_HF	Lfnu	Hfnu
Mood	0.497*	−0.367*	−0.331*	−0.420*	0.420*

**Note:**

* Significant correlation at 0.001 (bilateral).

**Table 5:** Multiple regression equation to explain morning mood as a function of morning HRV (*n* = 123 recordings).

Dependent variable	Independent variable	Non-standardised equation	Standardized coefficients (β)	R^2^	Sig.
Mood	RRmean SDRR Hfnu	Mood = –1.842 + (0.007 * Rrmean) – (0.026 * SDRR) + (0.034 * Hfnu)	0.515 (Rrmean) −0.381 (SDRR) 0.229 (Hfnu)	0.492*	<0.001

**Note:**

* Effect size: f^2^ = 0.968 (large > 0.35); Statistical power: *π* = 1.00 (for *n* = 123), three predictors and R^2^ = 0.492.

## Training RPE and Power data

Table 6 shows a significant correlation between the internal and the external load variables measured in the study, and in particular between the subjective RPE and the power data (NP, TSS and IF; *p* < 0.001 in all cases). In this way, a higher training power, meaning a higher training load, is reflected in a higher perfection of effort.

**Table 6:** Pearson correlation coefficients (r) between RPE and external load parameters in the same session (*n* = 66 recordings).

	NP	TSS	IF
RPE	0.553*	0.531*	0.545*

**Note:**

* Significant correlation at 0.001 (bilateral).

## Discussion

This study analysed the relationship between morning mood and HRV with regards to training load variables from the previous day. The association between morning mood and HRV on a given day, and between external and internal load variables was also studied. Correlation analyses were used to find association between variables and regression analyses was performed to try to explain mood and HRV as a function of training parameters from the previous day, and for explaining mood as a function of resting HRV parameters in the same morning session the day after training.

### Yesterday’s training correlates with this morning’s HRV

The current study indicated a relationship between morning HRV and training from the previous day. Specifically, Table 3 showed an inverse relationship between IF and HFnu, and a positive relationship between IF and LF/HF. Figure 1 illustrates, for Participant 1, the joint evolution over a month of the transformed parameters HFnu and IF. It can be seen, for example, that both pieces of data have an upward trend between 15-Jul and 23-Jul, a downward trend between 31-Jul and 4-Aug, and an opposite trend from 7-Jul to 8-Jul. The remaining training parameters under study, including NP and TSS, followed the same pattern as IF (*p* < 0.01), but with a lower significance (*p* < 0.05). Since Hfnu is considered a modulation index of the ANS parasympathetic branch (Burr, 2007), the results suggested that a higher training load one day (higher IF) correlated negatively with the activation of the vagal nerve the following morning, in favour of the SNS. This is in line with other studies, showing that parasympathetic power, indicated by HF, is able to reflect the recovery status hours after training (Chen et al., 2011; Seiler, Haugen & Kuffel, 2007), and that increased exercise intensity and/or duration cause delayed recovery of nocturnal cardiac autonomic modulation (Myllymäki et al., 2012).

As mentioned below (see Future Research), it would be interesting to track HFnu at regular hours and days post-exercise to help determine whether training was well-tolerated by the athlete, and in particular, to identify at what point the ANS switched from a sympathetic to a parasympathetic dominance. This is because the speed at which ANS recovers after exercise seems to be determined by fitness levels, with more rapid recovery in highly trained than in trained subjects after high-intensity exercise (Seiler, Haugen & Kuffel, 2007). Also, an increase of the parasympathetic branch following exercise is suggested to indicate the readiness of the ANS to take on strain again (Kiviniemi et al., 2007; Mueck-Weymann, Janshoff & Mueck, 2004) and is associated with improved athletic performance (Buchheit et al., 2010; Garet et al., 2004).

### Yesterday’s training correlates with this morning’s mood

Results showed an inverse relationship between IF and mood, so that the tougher one training session, the lower the mood of the following day (Table 4). This is contrary to the popular belief and most research on the topic, which argues that that exercise improves mood. However, such belief and research look at mood minutes, not days, after physical activity (Hansen, Stevens & Richard Coast, 2001; Lane & Lovejoy, 2001; Walters & Kearsley, 1993). Research looking at wellness scores the following day of exercise concluded that the greater the training load, the worse the wellness measures (fatigue, sleep quality, soreness and stress) (Buchheit et al., 2013). This goes in line with the results in Table 3. Other studies looking at mood over longer periods of time indicate that mood is affected by intense physical training in a dose-dependent manner, so that mood disturbances, as measured by the Profile of Mood States (POMS), increase in athletes during high-volume training sessions, in proportion to the training stimulus, and return to baseline during recovery (Gross et al., 2016; Purvis, Gonsalves & Deuster, 2010). At the same time, an impaired mood state and subjective complaints have mostly been described as sensitive and early markers of overtraining (Morgan et al., 1988; Urhausen & Kindermann, 2002), but not in all cases (Walters & Kearsley, 1993). While there is no clear way to define or diagnose overtraining, it is mostly characterised by ‘sports-specific’ decrease in performance, accompanied disturbances in mood state, including increase in fatigue, confusion, tension, depression, anxiety and lack of motivation and irritability, amongst others, persisting despite a period of recovery lasting several weeks or months (Meeusen et al., 2013; Stone et al., 1991; Walters & Kearsley, 1993).

In the present study it was found that the tougher one training session, the lower the mood of the following day, but it cannot be stated whether the change in mood was due to the intensity level of the training sessions nor from a state of overtraining. However, this is certainly a topic to be explored in future lines of research. Understanding short and long-term changes would be of interest to establish under which circumstances mood may be reliable and efficient to monitoring overtraining.

### Morning mood as a function of HRV

Findings in the current study indicate a relationship between morning mood and HRV, consistent with the idea that emotions that humans experience are associated with varying degrees of physiological arousal (Levenson, 2003). It was found that RRmean, SDNN and HFnu, in conjunction, explain 49.2% of mood variability of the same day (Table 5). Table 4 showed that mood correlated positively with RRmean and HFnu, and inversely with LFnu, LF/HF and SDRR (*p* < 0.005). Consistent with the idea of Hfnu as an index of the PNS (Burr, 2007), the results suggested that the more relaxed or recovered the nervous system, the more positive the emotional state. The findings are in line with other studies indicating a positive correlation between mood and HRV (Kindermann, 1986; Kleiger, Stein & Bigger, 2005; Koval et al., 2013; Lee, Wood & Welsch, 2003; Sakuragi, Sugiyama & Takeuchi, 2002). Geisler et al. (2010) supports the idea that HRV is associated with subjective well-being, whereas reduced HRV can be used as a predictive factor of the development of negative moods after situations such as deprivation of exercise (Weinstein, Deuster & Kop, 2007). However, it is interesting to note that most studies that found a significant correlation between mood and HRV were exclusively considering positives moods, including vigour and situations that trigger laughing, instead of negative moods (Sakuragi, Sugiyama & Takeuchi, 2002; Yoshino & Matsuoka, 2011). This could be due to the fact that positive emotions have a strong but transient effect on the ANS, while sadder moods have moderate but sustained effect (Sakuragi, Sugiyama & Takeuchi, 2002). Therefore, it would be interesting to study the relationship between morning HRV and mood not only on the same day, but also in consecutive days.

### RPE as an indicator of internal load

RPE, an internal load variable, correlated positively with external load variables such as NP, TSS and IF (*p* < 0.005). In this way, tougher workouts (higher power output) are reflected in higher RPE, supporting RPE as a significant measure of strain. This is consistent with other studies showing that session-RPE can be considered a valid, reliable and consistent indicator of global internal load (Coutts et al., 2003; Haddad et al., 2017; Impellizzeri et al., 2004; Impellizzeri, Rampinini & Marcora, 2005). This method was initially proposed by Foster et al. (1996) for monitoring internal training load in endurance athletes, but more recently is has also been proven to be applicable to other sports and physical activities with both men and women of different age and among different levels of experience (Haddad et al., 2017; Lupo, Ungureanu & Brustio, 2020; Wallace, Slattery & Coutts, 2009).

### Limitations

The main limitation of the current study is that despite having a number of recordings of *n* = 123 for Morning data and *n* = 66 for Training data, it only corresponds to five cyclists. A larger number of participants would be required in order to minimize individual differences and to increase the percentage of variability explained by the models of correlations and regression. Moreover, the lack of repeated measures fails to detect changes in HR and mood across different training situations, and future studies could include a follow-up assessment to capture the effects of different situations on the athletes, as proposed by Olmedilla et al. (2018). Another limitation of the study is the use of a non-standardized scale to measure morning mood. A reduced relationship between mood in regard to HRV and/or performance could come from the poor measurement reliability of such scale. Other scales might be more appropriate for monitoring mood, such as the POMS (Wallace, Slattery & Coutts, 2014). Finally, HRV, mood states and performance are variables that can be influenced by factors unrelated to training (Ortigosa-Márquez et al., 2017), such as food and drinks, medications, stress, etc. (Rodas et al., 2008). It would be interesting to record and analyse how these factors may interact with the parameters analysed and affect training.

### Future research

The aim of this research is geared towards developing a predictive system to assess training adaptations, which could help guide load distribution and detect and prevent overtraining. For such a system, and to accurately track fitness and fatigue, it is proposed to use a combination of subjective and objective variables, both from psychophysiological data and from external and internal load parameters (Haddad et al., 2017). The current study indicates a relationship between variables of internal (RPE) and external (IF) training parameters, subjective psychological states (Mood) and objective physiological data (HRV), but the relationships could be further explored. In particular, it would be of interest to look into the variations of HRV and mood at different points in time (hours and days) following training. This is because, as mentioned above, the relationship between training and HRV, or training and Mood, varies as time goes by. Tracking changes in HRV and mood in the long-term is also relevant because fitness has a longer time constant than physical and mental fatigue, meaning it asymptotes at a higher level and a later time (Morton, Fitz-Clarke & Banister, 1990), so only variations in HRV and/or mood over several weeks, rather than consecutive days, can help interpret changes in training. The study of long-term tendencies of HRV and mood as a determinant of training has already been proposed (Buchheit et al., 2010; Plews et al., 2013), but more research is still needed to consider the effectiveness of these variables for assessing training adaptation.

## Conclusions

An array of studies has recently shown an association between HRV and other internal and external measures of performance, performance and emotional states, and HRV and emotional states. In the current article, the aim was to explore the relationship between training of one day and general mood and HRV parameters of the following morning. The relationship between HRV and mood, and between internal and external approached to measure training, was also explored.

After recording morning HRV, morning mood, and training data of five amateur road cyclists during a 6 weeks period, the main results indicated a relationship between morning HRV at rest and training, so that training of one day correlated with HFnu of the following morning, with tougher training sessions (higher IF) leading to lower morning HFnu and higher Lfnu and LF/HF, suggesting reduced PNS in favour of SNS activity. Higher IF sessions also resulted in lower moods on the following morning, indicating a relationship between training and mood on consecutive days. The study also found that mood can be explained as a function of HRV at rest, such that the higher HFnu on a given day, the more positive the mood. Finally, the study found a significant correlation between external variables of training, including TSS and IF, and the internal RPE, supporting the role of subjective measures as viable indicators of training load.

Overall, the study explored the relationship between indicators of morning psychophysiological state of cyclists and training. Despite the need for further studies, it is advocated the potential of combining HRV, mood markers and internal and external load training data to provide insights into the pychophysiological state of the individual, their ability to improve performance, and the ability to detect and prevent overtraining.

## Supplemental Information

10.7717/peerj.13094/supp-1 Supplemental Information 1 Raw data.

## Additional Information and Declarations

### Data Availability

The following information was supplied regarding data availability: The raw measurements are available in the Supplemental File.
